# Preparation and Rheological Properties of Xanthoceras Sorbifolia Bunge Oil-Based Lubricating Oil Based on Ring-Opening Esterification Modification and Nano-C_14_MA/MMT Synergistic Strengthening

**DOI:** 10.3390/molecules30183830

**Published:** 2025-09-21

**Authors:** Zexin Li, Kai Zhang, Haoyue Wang, Tao Hou, Zhuoyi Lv, Wencong Li, Zhenpeng Wang, Yinan Hao

**Affiliations:** 1 College of Material Science and Art Design, Inner Mongolia Agricultural University, Hohhot 010010, China; 2 National Forestry Grassland Engineering Technology Research Center for Efficient Development and Utilization of Sandy Shrubs, Inner Mongolia Agricultural University, Hohhot 010010, China

**Keywords:** Xanthoceras sorbifolia oil-based lubricant, additives, esterification, viscosity–temperature performance, rheological properties

## Abstract

Lubricating oil plays a critical role in protecting mechanical systems. Driven by sustainable development strategies, the development of high-performance, biocompatible green lubricants has become an urgent industry need. Biomass resources, characterized by wide distribution, renewability, and environmental friendliness, represent ideal raw materials for replacing petrochemical-based lubricants. In this study, renewable Xanthoceras sorbifolia oil was utilized as the feedstock. Branched modification was achieved via ring-opening esterification using 2-ethylhexanol (2-EH) as the modifier and tetrafluoroboric acid (HBF_4_) as the catalyst. This epoxidation-branching modification process was synergistically combined with Nano-C_14_MA/MMT treatment. This approach significantly reduced high-temperature kinematic viscosity loss while maintaining excellent low-temperature flow properties, resulting in an Xanthoceras sorbifolia oil-based lubricant with outstanding viscosity–temperature performance and low-temperature fluidity. At a Nano-C_14_MA/MMT mass ratio of 0.3 wt% of the base oil, the lubricant demonstrated superior wide-temperature performance: KV_40_ = 424.1 mm^2^/s, KV_100_ = 50.8 mm^2^/s, VI = 180.8. The SP was reduced to −43 °C, exceeding the performance requirements of V-class environmentally friendly lubricants (e.g., synthetic ester oils). Furthermore, the coefficient of friction (COF) was 0.011 and the anti-wear scar diameter (AWSD) was 0.44 mm, indicating lubrication performance significantly superior to SN-class lubricants (specifications: COF < 0.12, AWSD < 0.50 mm).

## 1. Introduction

In recent years, significant progress has been made in the research and development of open-loop esterification technology for the production of plant-based lubricants. Plant-based lubricants have garnered significant attention due to their renewability, biodegradability, and environmental friendliness; however, their poor low-temperature flow properties and oxidative stability have limited their widespread application [1,2,3,4,5]. Studies have shown that plant oil-based lubricants modified by ring-opening esterification exhibit excellent oxidative stability, low-temperature flow properties, and tribological performance, capable of meeting lubrication requirements under various operating conditions [6,7,8,9,10]. Global lubricant consumption reached 49.1 million metric tons in 2020, maintaining a 3.2% average annual growth rate from 2014–2017 [11,12,13,14,15]. As the dominant consumer, China’s lubricant usage reached 6.6–6.9 million metric tons in 2023, with 5% average annual growth over the preceding five years. Traditional mineral-based oils still comprise > 90% of lubricant feedstocks [16,17]. Derived primarily from petroleum refining, their excessive consumption strains energy resources. Notably, olefin compounds in mineral oils exhibit biological toxicity. Leakage during production, storage, transportation, or operational loss contaminates aquatic and terrestrial systems through multiple pathways, posing severe threats to ecological balance [18,19].

Functional additives serve two primary roles in lubricating oil systems: compensating for base oil performance deficiencies and imparting specialized functional properties [20,21,22]. To satisfy application-specific requirements, scientifically proportioned additive-base oil combinations can be formulated for particular operational conditions [23,24,25,26]. Current mainstream additives comprise five categories: antioxidants (delaying oil aging), pour point depressants (enhancing low-temperature fluidity), friction modifiers (improving tribological performance), defoamers (suppressing foam formation), and emulsifiers (regulating oil–water interfacial behavior) [27,28]. Synergy between base oil refining technology and functional additives significantly enhances lubricant performance [29,30,31,32,33]. Concurrently, the emergence of bio-based and synthetic lubricants has accelerated the industry’s transition toward sustainability. Modern lubricants typically contain >90% base oil and <10% additives. The base oil governs fundamental properties like lubrication and heat dissipation, while additives extend application boundaries through functional enhancements, including oxidation inhibition, extreme pressure resistance, and anti-wear capabilities [12,34]. Collectively, they establish an integrated ‘carrier–function enhancement’ system capable of meeting complex operational demands [35,36,37].

To enhance low-temperature flow properties, achieve V-class standards, and minimize high-temperature kinematic viscosity loss in lubricants, we investigated the epoxidation of unsaturated fatty acids in Xanthoceras sorbifolia oil using 2-ethylhexanol with tetrafluoroboric acid (HBF_4_) catalysis. Subsequent ring-opening esterification incorporated Nano-C_14_MA/MMT into the lubricant system for synergistic performance enhancement. This process yielded a highly branched Xanthoceras sorbifolia-based lubricant with exceptional viscosity-temperature performance (VI > 180) and superior low-temperature fluidity (SP < −40 °C), significantly broadening its operational temperature range. The optimized process was validated through molecular structure analysis via FTIR, while rheological properties—including viscosity–temperature behavior, pour point, and thermal stability—were characterized using kinematic viscometry, pour point testing, rheometry, thermogravimetric analysis (TGA), and four-ball tribometry.

## 2. Results

**Figure 1:**
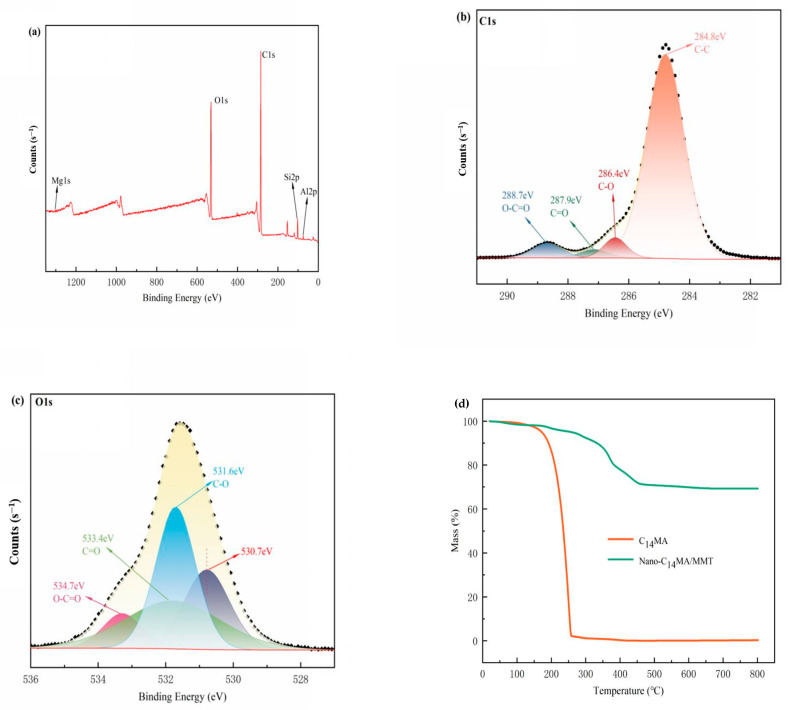
(**a**–**c**) XPS spectrum of Nano-C_14_MA/MMT and high-resolution XPS spectra of C1s, O1s; (**d**) TG curves of Nano-C_14_MA/MMT.

As shown in Figure 1a, the photoelectron peaks of Si(2p), Al(2p), and Mg(2p) in Nano-C14MA/MMT were observed at 103 eV, 75 eV, and 50 eV, respectively. The C1s XPS spectra of Nano-C_14_MA/MMT (Figure 1b) reveal peaks corresponding to C-C, C=O, C-O, and C-O-C at 284.8 eV, 287.9 eV, 286.4 eV, and 288.7 eV, respectively. Similarly, the O1s spectra (Figure 1c) display peaks for C-O, C=O, and C-O-C at 531.6 eV, 533.4 eV, and 534.7 eV, respectively, alongside a lattice oxygen peak (from Si-O, Mg-O, and Al-O bonds) at 531.1 eV. Combined with FTIR analysis, these results confirm the successful grafting of C_14_MA onto MMT, verifying the composite’s synthesis. Pure C14MA undergoes pyrolysis primarily between 170 °C and 250 °C (Figure 1d). MMT—a layered silicate clay mineral composed of silicon–oxygen tetrahedra and aluminum–oxygen octahedra—exhibits high thermal stability and chemical resistance, delaying polymer pyrolysis by adsorbing gaseous and small-molecular products. Consequently, the pyrolysis temperature range for Nano-C_14_MA/MMT broadens and shifts upward to 200–430 °C.

The Nano-C_14_MA/MMT composite exhibits a uniformly dispersed lamellar structure without agglomeration (Supplementary Figure S1), ensuring stable dispersion in lubricants. MMT surfaces display irregularly stacked folds that facilitate C_14_MA attachment. Particulate features above wrinkles indicate uniform C_14_MA coating on MMT, yielding a surface roughness attributed to strong MMT-C_14_MA adhesion. FTIR analysis (Figure S2a) revealed the disappearance of the C=C stretching band (1640 cm^−1^) and MMT-associated bands (Si–O: 1030 cm^−1^; –OH: 3400 cm^−1^), with concurrent emergence of a C–O–C stretching band (1250 cm^−1^), confirming successful polymerization. XRD patterns (Figure S2b) showed attenuated MMT diffraction peaks, indicating lamellar disordering through C_14_MA polymer intercalation.

Figure S3 demonstrates the appearance of an epoxy group (–O–) at 830 cm^−1^ and the disappearance of the C=C band at 1660 cm^−1^ in epoxidized Xanthoceras sorbifolia oil (EXSBO), confirming successful epoxidation. Furthermore, in 2-ethylhexanol-modified EXSBO, the disappearance of the epoxy peak (830 cm^−1^), appearance of C–O–C at 1036 cm^−1^, and broadened –OH band at 3400 cm^−1^ collectively verify successful modification. The broad shape of the –OH band (3400 cm^−1^) indicates hydrogen-bonded hydroxyl groups, enhancing polarity and improving adsorption on metal friction surfaces through physical/chemical lubricating films, thereby reducing friction and wear.

**Figure 2:**
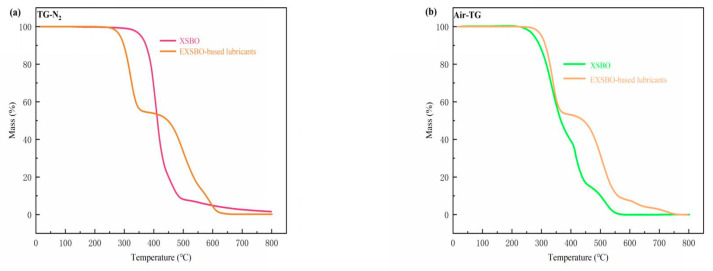

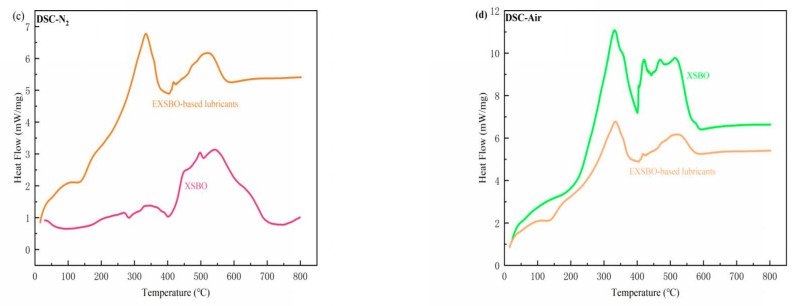
(**a**,**c**) TG-DSC curves of XSBO and EXSBO base lubricants under nitrogen atmosphere; (**b**,**d**) TG-DSC curves of XSBO and EXSBO base lubricants in an air atmosphere.

Figure 2a,b reveals that Xanthoceras sorbifolia oil (XSBO) maintains constant mass from 0–300 °C under both air and nitrogen atmospheres, with no mass loss. Between 300 and 500 °C, thermal cleavage of C–C and C–O bonds in XSBO triggers significant degradation. Similarly, EXSBO-based lubricants show stable mass at 0–250 °C regardless of atmosphere but exhibit pronounced mass loss at 250–600 °C under nitrogen. DSC curves (Figure 2c,d) indicate gradual heat flow increases for both oils at 0–250 °C, reflecting rising heat absorption with temperature. EXSBO-based lubricants (Nano-C_14_MA/MMT) display higher heat flux than XSBO. At ≈300 °C, distinct endothermic peaks—correlating with TG data—signify molecular chain scission. The higher endothermic peak for EXSBO confirms greater energy absorption during degradation. Between 400 and 550 °C, minor exothermic peaks in both samples arise from secondary reactions (e.g., recombination/polymerization of pyrolytic fragments). Throughout the temperature range, EXSBO-based lubricants demonstrate superior thermal stability via sustained higher heat flux.

**Figure 3:**
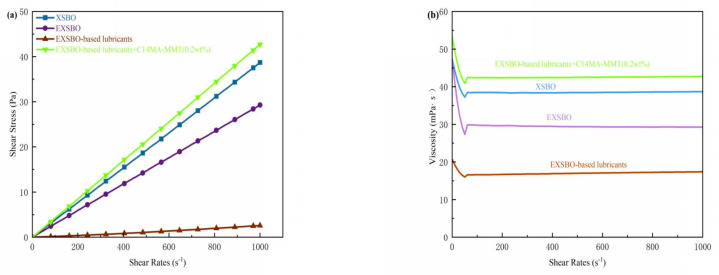
(**a**) Shear stress-rate curves for XSBO and EXSBO-based lubricants (0.3 wt% Nano-C14MA/MMT); (**b**) Shear stress-viscosity relationship of XSBO and EXSBO-based lubricants (0.3 wt% Nano-C14MA/MMT).

Figure 3a depicts the shear stress–shear rate curves for Xanthoceras sorbifolia oil (XSBO) and EXSBO-based lubricant (0.3 wt% Nano-C_14_MA/MMT), while Figure 3b shows the shear stress–viscosity relationships for both oils. Linear shear stress–shear rate profiles across temperatures confirm Newtonian behavior, where viscosity depends solely on temperature. This characteristic validates their suitability as lubricants.

Table 1 summarizes kinematic viscosity data. The EXSBO-based lubricant (0.3 wt% Nano-C_14_MA/MMT) exhibits kinematic viscosities of 424.1 mm^2^/s at 40 °C (KV40) and 50.8 mm^2^/s at 100 °C (KV100), with a viscosity index (VI) of 180.7. This elevated VI reflects reduced high-temperature viscosity loss, improved low-temperature fluidity, and enhanced overall stability. The lubricant’s freezing point decreases to −43 °C due to suppressed molecular entanglement (via C_14_-MA branching) and diminished polar aggregation, optimizing the viscosity–temperature behavior. Montmorillonite forms a shear-resistant nanonetwork, while C_14_-MA chains inhibit wax crystallization; their interfacial synergy prevents viscosity–temperature deterioration through molecular structure optimization and physicochemical coupling.

Table 1 confirms that the flash point of modified Xanthoceras sorbifolia oil-based lubricant exceeds 264 °C, which is attributable to ring-opening esterification, enhancing molecular branching and weight. The lubricant’s freezing point (−43 °C) is 25 °C lower than unmodified oil due to Nano-C_14_MA/MMT nanoparticles: (1) acting as nucleation sites that refine wax crystal size/distribution, delaying macroscale network formation; and (2) dispersing to increase inter-crystal distances while adsorbing onto crystal surfaces, suppressing growth/aggregation [38]. This dual mechanism impedes large-network crystallization, further depressing the freezing point.

**Figure 4:**
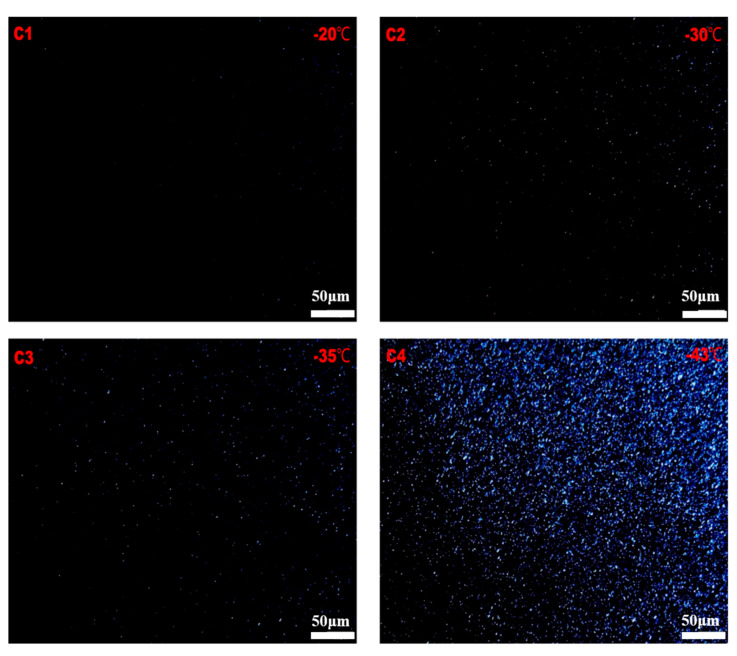
Polarizing microscope images of XSBO and EXSBO-based lubricants (0.3 wt% Nano-C_14_MA/MMT) crystallized at different low temperatures.

Polarized optical microscopy (Figure 4) reveals the low-temperature crystallization morphology of EXSBO-based lubricant (0.3 wt% Nano-C_14_MA/MMT). The composite achieves a pour point of −43 °C–19 °C lower than unmodified Xanthoceras sorbifolia oil. As the temperature decreases from −5 °C to −37 °C, wax crystal density increases. Crucially, the lubricant exhibits fewer crystals than XSBO (Figure S4, O1–O4) and transitions needle-like crystals into uniformly distributed microspheres. This ordered morphology persists during cooling to −41 °C despite gradual crystal growth. Nano-C_14_MA/MMT thus functions as an effective pour point depressant by restructuring crystallization dynamics.

As shown in Table 2, when the addition of Nano-C_14_MA/MMT is 0.3 wt%, the friction coefficient of the prepared lubricating oil decreases significantly compared to that of tung oil. The reduction in the friction coefficient is 0.011. Based on the relationship between the friction coefficient and the type of friction, it can be concluded that the friction type of this lubricating oil is mixed friction [39]. This regime reduces interfacial resistance, minimizes wear, and prolongs component longevity under equivalent loads. The enhancement arises from Nano-C_14_MA/MMT’s dual action: increasing kinematic viscosity (Table 1) and strengthening metal surface adsorption via polar groups [40]. Consequently, a robust protective oil film forms on friction surfaces, enhancing lubrication efficiency.

Table 3 shows a wear scar diameter of 0.44 mm for EXSBO-based lubricant (0.3 wt% Nano-C_14_MA/MMT), which is significantly lower than XSBO. This reduction stems from nanoparticles filling surface microcracks, repairing damage, and forming protective tribofilms. Simultaneously, they reinforce oil film strength and stability under high loads while imparting extreme-pressure resistance that minimizes wear under severe conditions. Enhanced thermal stability further mitigates high-temperature performance degradation.

**Figure 5:**
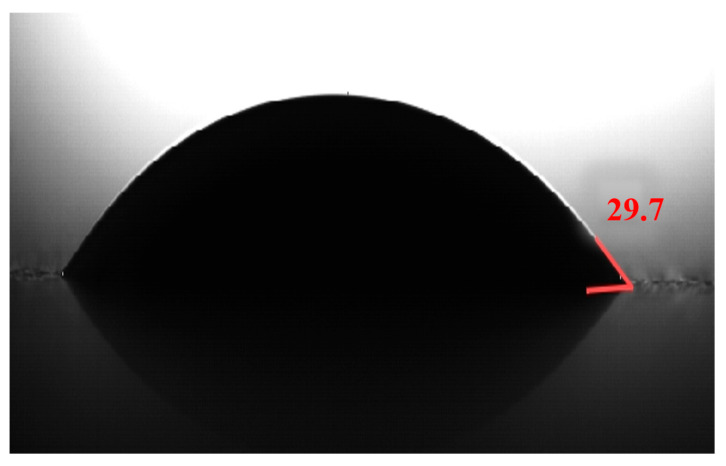
The contact angle of EXSBO-based lubricants (0.3 wt% Nano-C_14_MA/MMT).

Contact angle analysis (Figure 5) quantifies lubricant surface compatibility and wettability, revealing a 29.7° angle for modified Xanthoceras sorbifolia oil. Nano-C_14_MA/MMT nanoparticles reduce surface tension through dispersion, enhancing wettability and promoting uniform surface coverage. Conversely, ring-opening esterification diminishes polar groups (e.g., hydroxyls), lowering surface energy and increasing contact angle. The contact angle of XSBO is 34.5°. The net angle reduction in EXSBO-based lubricant (0.3 wt% Nano-C_14_MA/MMT) demonstrates superior wettability versus XSBO, resulting from competing nanostructural modifications and polarity adjustments.

## 3. Materials and Methods

### 3.1. Chemicals and Materials

All chemicals were used as received without further purification. These included Xanthoceras sorbifolia oil, montmorillonite (MMT; Macklin, Shanghai, China), cetyltrimethylammonium bromide (CTAB; Macklin Shanghai, China), tetradecyl methacrylate (C14MA; Macklin Shanghai, China), benzoyl peroxide (BPO; Macklin Shanghai, China), petroleum ether, N,N-dimethylformamide (DMF; Macklin Shanghai, China), hydrogen peroxide (Macklin Shanghai, China), sulfuric acid (98%), anhydrous sodium carbonate, 2-ethylhexanol (2-EH; Macklin Shanghai, China), and fluoroboric acid (HBF4; Macklin Shanghai, China). Deionized water was employed throughout the study.

### 3.2. Preparation of Montmorillonite/Tetradecyl Methacrylate Surfactant (Nano-C_14_MA/MMT)

Organically modified montmorillonite (OMMT) [41,42] was prepared via CTAB treatment at a 20% CTAB-to-MMT mass ratio. The Nano-C_14_MA/MMT pour point depressant was synthesized through in situ free radical polymerization. Specifically, 1.43 mmol OMMT was ultrasonically dispersed in 0.65 mol N,N-dimethylformamide (DMF) at room temperature for 1 h. This suspension was transferred to a 250 mL flask, purged with N_2_ for 30 min, then charged with 0.2 mol C_14_MA and 0.6 mmol benzoyl peroxide (BPO) [43]. The reaction system underwent in situ polymerization at 80 °C under N_2_ atmosphere for 4 h. The resultant Nano-C_14_MA/MMT depressant [7] was isolated by ethanol washing followed by 24 h vacuum drying.

### 3.3. Preparation of Ring-Opening Esterification of Modified Xanthoceras Sorbifolia Oil-Based Lubricant Base Oil

**Scheme 1:**
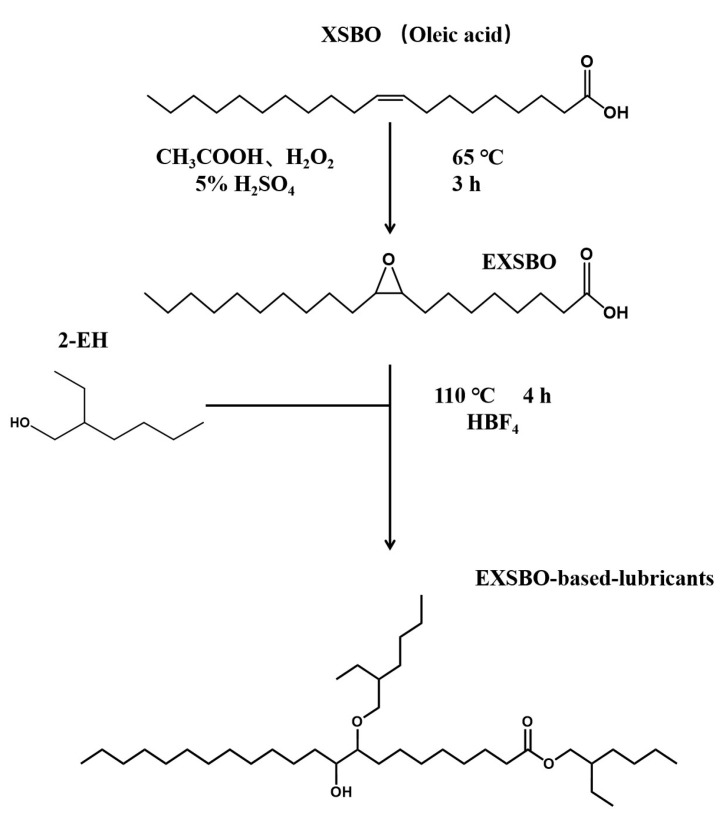
The mechanism diagram of ring-opening esterification reaction.

As shown in Scheme 1, capitalizing on the high unsaturated fatty acid content of Xanthoceras sorbifolia oil, toluene was employed as an organic phase diluent to reduce mixture viscosity. The epoxidation proceeded at 60 °C for 6 h with sulfuric acid stabilization to prevent excessive double-bond oxidation. The resultant organic phase underwent sequential washing with 2 wt% sodium carbonate solution (neutralizing residual acid) and warm water (removing excess carbonate), followed by vacuum distillation at 60 °C to remove toluene and residual water, yielding epoxidized Xanthoceras sorbifolia oil (EXSBO). Subsequent branching modification utilized EXSBO with 2-ethylhexanol (2-EH) as the chain modifier and tetrafluoroboric acid (HBF_4_) catalyst at mass ratios EXSBO:2-EH = 5:9 and HBF_4_:EXSBO = 5 wt%. The reaction was conducted at 110 °C for 4 h. The resulting lubricant was purified through repeated 2 wt% sodium carbonate solution and warm water washes. Subsequently, residual moisture, excess 2-EH, and BF_3_-alcohol complexes were removed by rotary evaporation at 110 °C under vacuum conditions.

### 3.4. Preparation of Xanthoceras Sorbifolia Oil-Based Lubricating Oil Based on Synergistic Enhancement of Ring-Opening Esterification Modification and Nano-Depressant

Different mass fractions of Nano-C_14_MA/MMT (0 wt%, 0.05 wt%, 0.1 wt%, 0.15 wt%, 0.2 wt%, 0.25 wt%, 0.3 wt%) were added to EXSBO-based lubricants, dissolved and dispersed at 60 °C, and cooled to room temperature to obtain modified Xanthoceras sorbifolia oil-based lubricants (EXSBO-based lubricants [Nano-C_14_MA/MMT]).

### 3.5. Material Characterization

The phase structure and lattice constants of all samples were analyzed by X-ray diffraction (XRD). The scanning angle range was 5° to 90°, and the rate was 2°/min. The ALPHA type Fourier transform infrared spectrometer was used for testing. The test conditions were as follows: scanning speed 32 times/s, resolution 4 cm^−1^, test wave number range 400–4000 cm^−1^. The morphology of the samples was observed by a Regulus8220 scanning electron microscope (Hitachi, Tokyo, Japan). Additive samples were analyzed by XPS (Escalab 250 Xi, Thermo Fisher Scientific, Waltham, MA, USA). The STA449F3 synchronous thermal analyzer (NETZSCH Analyzing & Testing, Selb, Germany) was used to record the quality change of the oil.

### 3.6. Xanthoceras Sorbifolia Oil-Based Lubricating Oil Performance Test

The rheological properties, such as shear rate–shear stress, shear rate–viscosity, and viscosity–temperature, were tested by an AR2000 CS rheometer (TA Instruments, New Castle, DE, USA). The MRS-1J four-ball friction and wear tester (Jinan Shunmao Testing Instruments Co., Ltd., Jinan, China) was used to carry out the friction and wear test on the sample. The precision bearing steel ball with a diameter of 12.7 mm was selected as the friction ball, and the load was 392 N. After grinding for 30 min at a rate of 1200 r/min, the lubrication performance of the lubricating oil was measured.

### 3.7. Test of Freezing Point, Pour Point, and Flash Point

First, the sample water bath was heated to 50 ± 1 °C, then the test tube containing the sample and thermometer was placed at room temperature and naturally cooled to 35 ± 5 °C, and then the test tube was placed in a cold bath. When the temperature of the cold bath reached the set temperature, the cold bath was tilted 45 degrees and kept for one minute, and then the sample test tube was taken out to observe whether the liquid surface moved. When the liquid level position remained unmoved, the test temperature was increased by 4 °C, and the test was repeated. When the test temperature maintained the test tube’s liquid level (containing the sample and thermometer), and the liquid level moved when the test temperature increased by 2 °C, the temperature of the liquid level is the freezing point of the sample. The sample was placed in the test cup up to the specified mark. The sample temperature was rapidly increased, then a slow, constant rate of heating was maintained as the expected flash point was approached. At predetermined temperature intervals, a small test flame (igniter) was introduced through the ignition port into the cup. The lowest temperature at which the vapor above the sample flashes is the flash point of that sample.

### 3.8. Low-Temperature Crystal Morphology and Surface Wettability Test

Sample imaging was performed using a Leica DM 2500P polarizing microscope (Leica, Wetzlar, Germany). A 2 μL aliquot of each sample was deposited onto a glass slide and covered with an 18 × 18 mm cover slip prior to observation. The slides were placed in a cold storage and quickly frozen to −50 °C (20 °C/min) for 1 min. The sample temperature was then raised to a specified temperature (10 °C/min) and held for 5 min to record the ice crystal image. The contact angle of the lubricating oil was tested by a V5 contact angle measuring instrument.

## 4. Conclusions

In this study, lubricants derived from Xanthoceras sorbifolia oil (XSO) were synthesized through ring-opening esterification modification with synergistic enhancement using Nano-C_14_MA/MMT. The preparation involved sequential epoxidation, ring-opening esterification, and incorporation of Nano-C14MA/MMT as a pour point depressant. Comprehensive rheological characterization revealed that the EXSBO-based lubricant (0.3 wt% Nano-C_14_MA/MMT) exhibits kinematic viscosities of 424.1 mm^2^/s at 40 °C (KV40) and 50.8 mm^2^/s at 100 °C (KV100), with a viscosity index (VI) of 180.8, classifying it as a very high VI lubricant. The solidifying point decreased significantly to −43 °C, a 25 °C reduction relative to unmodified XSO, substantially broadening its operational temperature range and demonstrating superior viscosity–temperature behavior and low-temperature fluidity. Rheological performance exceeded Class V (synthetic ester-based) environmentally friendly lubricants. Thermogravimetric and differential scanning calorimetry (TG-DSC) analyses confirmed exceptional thermal and oxidative stability between 0 °C and 250 °C. Tribological testing yielded a coefficient of friction (COF) of 0.011 and an average wear scar diameter (AWSD) of 0.44 mm, indicating significantly enhanced lubrication compared to unmodified XSO and commercial SN-grade lubricants. A contact angle of 29.7° further validated excellent wettability and surface compatibility. Collectively, the developed lubricant exhibits outstanding rheological properties, thermal resilience, and tribological performance, demonstrating broad application potential.

## Figures and Tables

**Table 1:** Analysis of rheological properties of XSBO and EXSBO-based lubricants (Nano-C_14_MA/MMT).

Sample	Nano- C_14_MA/MMT (wt%)	KV_40_ (mm^2^/s)	KV_100_ (mm^2^/s)	VI	Solidifying Point (SP) (°C)	Flash Point (FP) (°C)
EXSBO-based lubricants (wt%Nano- C_14_MA/MMT)	0	76.2	19.2	110.3	−18	190
	0.05	116.2	21.7	128.2	−20	192
	0.1	161.7	25.5	133.7	−23	201
	0.15	235.3	31.8	136.3	−27	210
	0.2	293.3	36.5	141.1	−30	237
	0.25	375.4	44.2	153.8	−36	253
	0.3	424.1	50.8	180.7	−43	264
	0.35	399.2	46.2	177.2	−35	251

**Table 2:** Friction coefficient of EXSBO-based lubricants (0.3 wt% Nano-C_14_MA/MMT).

Specimen	COF
XSBO	0.099
EXSBO-based lubricants (0.3 wt% Nano-C_14_MA/MMT)	0.011

**Table 3:** The wear scar diameter of EXSBO-based lubricants with Nano-C_14_MA/MMT content of 0.3 wt%.

Specimen	1		2		3		AWSD (mm)
	X1	Y1	X2	Y2	X3	Y3	
**XSBO**	0.7	0.7	0.58	0.72	0.74	0.71	0.69
**EXSBO-based lubricants** **(0.3 wt% Nano-C_14_MA/MMT)**	0.43	0.45	0.41	0.42	0.48	0.45	0.44

## Data Availability

The original contributions presented in this study are included in the article/Supplementary Material. Further inquiries can be directed to the corresponding author.

## Supplementary Materials

The following supporting information can be downloaded at: https://www.mdpi.com/article/10.3390/molecules30183830/s1, Figure S1. The SEM images of Nano-C_14_MA/MMT. Figure S2. (a) The FT-IR Spectra of MMT, OMMT, C_14_MA, Nano-C_14_MA/MMT; (b) XRD patterns of MMT, Nano-C_14_MA/MMT, Nano-C_14_MA/MMT. Figure S3. The FT-IR spectra of XSBO, EXSBO and EXSBO-based lubricants. Figure S4. Polarizing microscope images of XSBO-based lubricants (0.25 wt% Nano-C_14_MA/MMT) crystallized at different low temperatures (−10 °C, −15 °C, −18 °C, −26 °C). Figure S5. The contact angle of XSBO and XSBO-based lubricants (0.2 wt% CMK-EC).
