# Supplementary material for: Preparation and Rheological Properties of Xanthoceras Sorbifolia Bunge Oil-Based Lubricating Oil Based on Ring-Opening Esterification Modification and Nano-C14MA/MMT Synergistic Strengthening
Source: Molecules. 2025 Sep 21;30(18):3830. doi: 10.3390/molecules30183830 (PMC12472505; doi:10.3390/molecules30183830)
Supplement: Supplementary file 1 [file molecules-30-03830-s001.zip › molecules-3775492-supplementary.pdf]

Preparation and Rheological Properties of Xanthoceras Sorbifolia Bunge Oil-Based Lubricating Oil Based on Ring-Opening Esterification Modification and Nano-C_{14}MA/MMT Synergistic Strengthening

Zexin Li [1,†], Kai Zhang [1,†], Haoyue Wang [1,†], Tao Hou [1], Zhuoyi Lv [1], Wencong Li [1], Zhenpeng Wang [1] and Yinan Hao [1,2,*]

[1] College of Material Science and Art Design, Inner Mongolia Agricultural University, Hohhot 010010, China
[2] National Forestry Grassland Engineering Technology Research Center for Efficient Development and Utilization of Sandy Shrubs, Inner Mongolia Agricultural University, Hohhot 010010, China
* Correspondence: nanyihao83@163.com
† These authors contributed equally to this work.

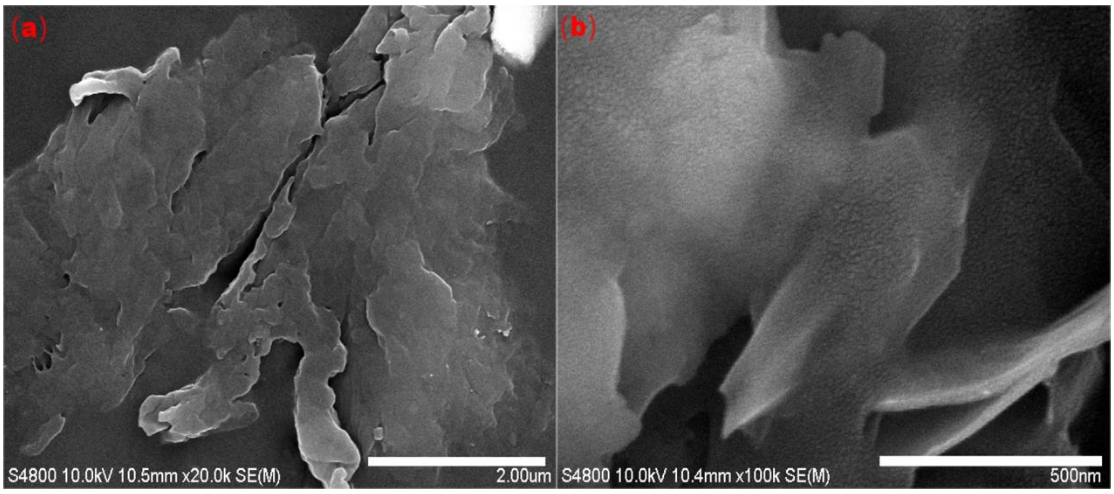

Figure S1.(a) Scanning Electron Microscope image of Nano-C14MA/MMT at 2 μ m; Scanning Electron Microscope image of Nano-C14MA/MMT at 500nm

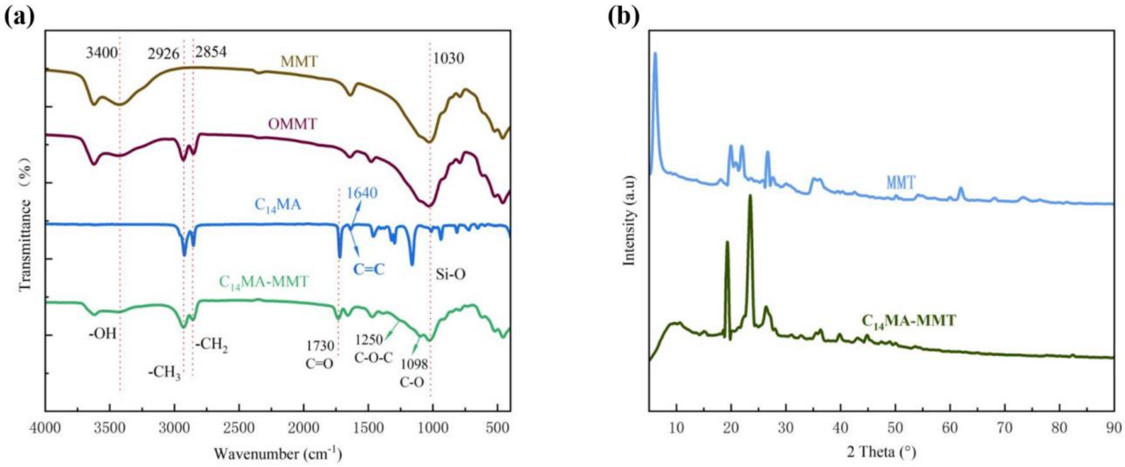

Figure S2.(a) The FT-IR Spectra of MMT, OMMT, $C_{14}MA$、Nano-$C_{14}MA$/MMT;(b)XRD patterns of MMT、Nano-$C_{14}MA$/MMT、Nano-$C_{14}MA$/MMT

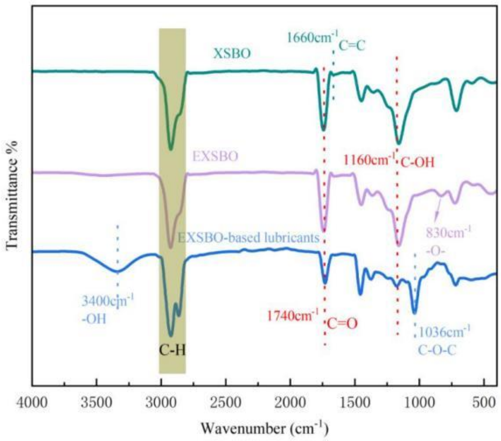

Figure S3. The FT-IR spectra of XSBO、EXSBO and EXSBO-based lubricants

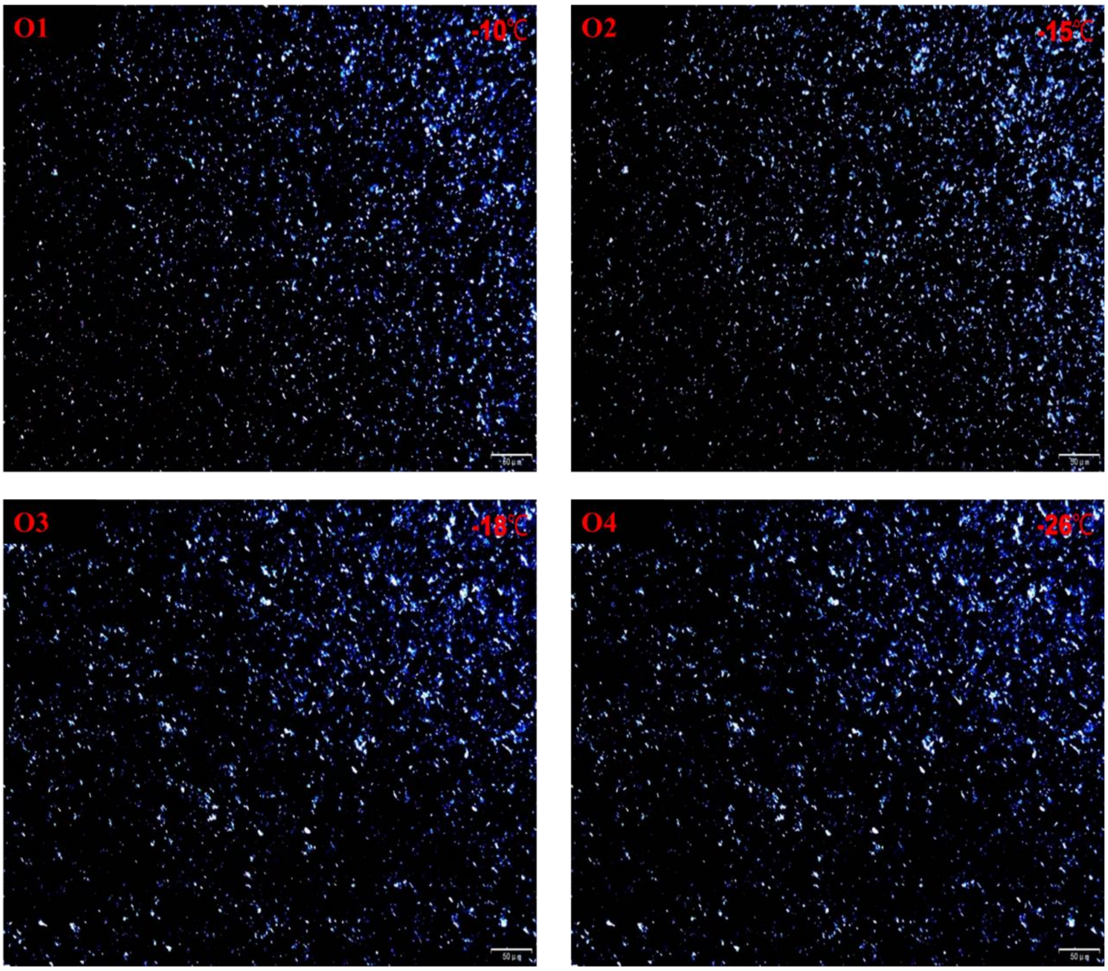

Figure S4. Polarizing microscope images of XSBO-based lubricants(0.25wt%Nano-C_{14}MA /MMT) crystallized at different low temperatures(-10 ℃、-15 ℃、-18 ℃、-26 ℃)

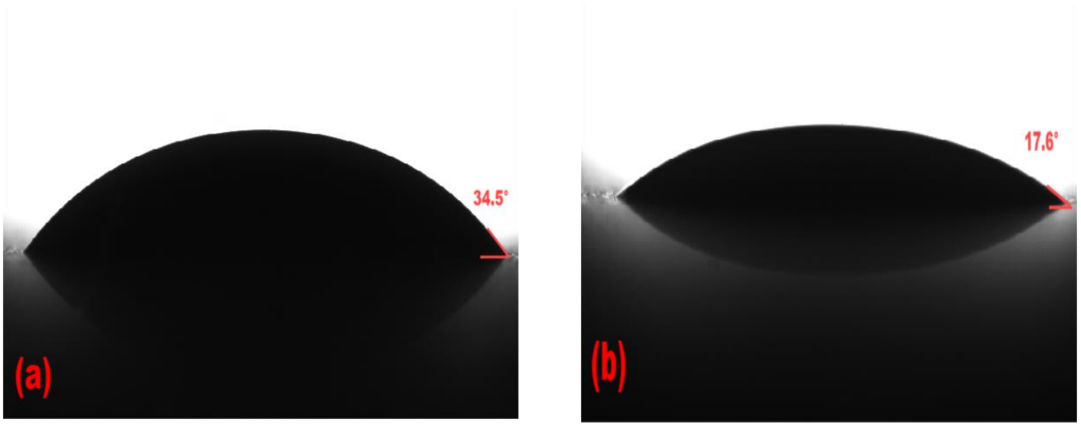

Figure S5. （a）The contact angle of XSBO;（b) The contact angle of XSBO-based lubricants(0.2wt%CMK-EC)
